# Establishment of an ELISA for detecting oocyst-derived *Toxoplasma gondii* infections in sheep

**DOI:** 10.3389/fvets.2025.1674011

**Published:** 2026-01-14

**Authors:** Xinran Peng, Tiantian Geng, Yu Wang, Fen Du, Junlong Zhao, Rui Fang, Bang Shen, Yanqin Zhou

**Affiliations:** 1 National Key Laboratory of Agricultural Microbiology, College of Veterinary Medicine, Huazhong Agricultural University, Wuhan, Hubei, China; 2 Hubei Center for Animal Disease Prevention and Control, Wuhan, Hubei, China

**Keywords:** ELISA, epidemiological investigation, oocyst infection, ovine toxoplasmosis, TGME49_2674106, *Toxoplasma gondii*

## Abstract

**Background:**

Ovine toxoplasmosis is a zoonotic disease that severely impacts the development of the sheep industry. The primary routes of *Toxoplasma gondii* infection in sheep are cyst infection and oocyst infection. However, current technologies are unable to distinguish between these two infection pathways.

**Methods:**

In this study, we initially screened eight proteins that are highly specifically expressed during the oocyst stage. Through Western blot (WB) analysis, we identified a protein (TGME49_267410) that could serve as a diagnostic antigen. Subsequently, we optimized the conditions for an indirect enzyme-linked immunosorbent assay (iELISA) using TGME49_267410. Based on these optimized conditions, we collected 1,350 sheep serum samples from various prefecture-level cities in Hubei Province and compared the detection results using both GRA1-iELISA and *Tg*267410-iELISA.

**Results:**

In this study, we successfully identified TGME49_267410 as a specific diagnostic antigen for *Toxoplasma gondii* oocyst-derived infections and established a *Tg*267410-based indirect enzyme-linked immunosorbent assay (*Tg*267410-iELISA) for antibody detection. This method exhibited excellent specificity with no cross-reactivity against ovine *Haemonchus contortus* infections, along with a low limit of detection and good stability of enzyme-linked plates. Serological testing of sheep serum samples from Hubei Province revealed an overall oocyst infection positive rate of 47.4%, where the positive rate in commercial fattening farms (39.9%) was significantly higher than that in breeding sheep farms (12.2%).

**Conclusion:**

To summarize, the *Tg*267410-iELISA established herein enables specific, sensitive, and stable detection of ovine *Toxoplasma gondii* oocyst-derived infections. This method facilitates the differentiation of infection routes and epidemiological surveillance of ovine toxoplasmosis in Hubei Province and beyond, providing a robust scientific foundation for optimizing targeted prevention and control strategies in sheep farms.

## 1 Introduction

Toxoplasmosis in sheep, resulting from infection with the protozoan parasite *Toxoplasma gondii*, represents a significant veterinary and economic concern. *T. gondii* is known for its broad host range, capable of infecting numerous vertebrate species, and is regarded as one of the most successful eukaryotic pathogens globally (1–6). Human seroprevalence of *T. gondii* averages about 30.0%, while infection rates among livestock, particularly sheep, range between 40.0 and 60.0%, underscoring the substantial zoonotic threat (7–9). In ovine species, *T. gondii* infection is a major cause of reproductive loss, associated with abortion rates of 20.0–30.0%, thereby imposing severe economic impacts on sheep production systems. In China, the overall seroprevalence in sheep flocks has been reported at 9.90%; however, regional disparities reveal a pronounced endemic pattern, with average infection rates exceeding 20.0% in North China, South China, and Southwest China (10). Although a commercial live vaccine (Toxovax) has been developed for ovine toxoplasmosis, its widespread application remains constrained by considerable safety issues (11, 12).

Diagnosis of *Toxoplasma gondii* infection remains challenging due to the nonspecific nature of its clinical manifestations, which often render clinical examination and necropsy findings inconclusive and prone to misdiagnosis (13). While accurate diagnosis generally depends on parasitological and serological techniques, standardized and commercially available diagnostic tools for ovine toxoplasmosis are still lacking. Among existing methods, the enzyme-linked immunosorbent assay (ELISA) offers distinct advantages, including high sensitivity and specificity, as well as the capacity for high-throughput testing of clinical samples within short timeframes (14). These characteristics make ELISA particularly suitable for field epidemiological studies and large-scale screening, garnering significant research interest. In efforts to improve serological detection, multiple studies have developed ELISA protocols using recombinant *T. gondii* antigens. Classical antigenic targets such as SAG1, SAG2, GRA1, GRA2, GRA6, and GRA7 have been extensively evaluated as diagnostic markers (15–20). Moreover, Hill et al. identified the sporozoite-specific embryogenesis-related protein (ERP) as a promising antigen capable of distinguishing infections mediated by oocysts (21).

*Toxoplasma gondii* is transmitted through various routes, but existing diagnostic techniques can only indicate whether sheep are infected with the parasite, without differentiating the specific route of infection. In this study, we aimed to develop a specific antibody ELISA method for detecting ovine infection with *T. gondii* oocysts by identifying proteins that are highly expressed during the oocyst stage but are lowly or non-expressed in other stages. This method, combined with the GRA1-iELISA diagnostic method previously established in our laboratory, can distinguish the specific routes of *T. gondii* infection in sheep. An epidemiological analysis of *T. gondii* infection routes in sheep in Hubei Province was conducted to identify the sources of infection. Based on these findings, customized prevention and control strategies for ovine toxoplasmosis can be developed, providing valuable insights for tracing the origins of the disease and implementing effective control measures in the sheep farming industry.

## 2 Materials and methods

### 2.1 Plasmids and parasite strains

The *Toxoplasma gondii* ME49 strain (preserved in the Parasitology Laboratory of Huazhong Agricultural University) was propagated in HFF cells (ATCC, USA) cultured in DMEM medium (Invitrogen, USA) supplemented with 2% fetal bovine serum (FBS). The *Escherichia coli* BL21 (DE3) strain used for expression was also preserved in the Parasitology Laboratory of Huazhong Agricultural University. The recombinant plasmid pET-28a was constructed and preserved in the same laboratory. The construction of the pET-28a-267410 plasmid was performed as follows: RNA was extracted from the tachyzoites of the *T. gondi*i ME49 strain, and cDNA was synthesized using the PrimeScript RT reagent Kit with gDNA Eraser (TaKaRa, Japan) (22). The coding sequence of 267410 was amplified using primers 267410 (CDS)-F and 267410 (CDS)-R (Table 1). The amplified fragment was then cloned into the pET-28a vector using the CloneExpress One Step Cloning Kit (Vazyme, China) to generate pET-28a-267410. The construct was confirmed by restriction enzyme digestion and sequencing and was subsequently transformed into *E. coli* BL21 (DE3) for inducible expression. The plasmids pET-28a-TGME49_313000, pET-28a-TGME49_316550, pET-28a-TGME49_281590, pET-28a-TGME49_292960, pET-28a-TGME49_242600, pET-28a-TGME49_271570, and pET-28a-TGME49_271580 were constructed using the same method (Table 1).

**Table 1:** Primers used in this study and their sequences.

Primer name	Sequence 5′–3′
pET-28a(CDS)-F	AACAAAGCCCGAAAGGAAGC
pET-28a(CDS)-R	GGATCCGCGACCCATTTGCT
313000-F	AGCAAATGGGTCGCGGATCCACGCAGCAAGTGGCAGTC
313000-R	TGGTGGTGGTGGTGCTCGAGCTTCTTCGTAGCATTCATACTGCTCG
316550-F	CAAATGGGTCGCGGATCCGAACATGTTGGTGACGCGAAG
316550-R	GGTGGTGGTGGTGCTCGAGCCAGTAATACCCACCGTAGCC
281590-F	CAAATGGGTCGCGGATCCATGGAAGAACCCGAACGCATC
281590-R	GGTGGTGGTGGTGCTCGAGCCAATAGTGGCGCCCCCAAAAG
292960-F	CAAATGGGTCGCGGATCCGAGGGACTGGCTCAATTTG
292960-R	GGTGGTGGTGGTGCTCGAGCTCTGTGTCATCTGTTTCTTCTG
242600-F	CAAATGGGTCGCGGATCCGGAGCTCGGACACTTGAAG
242600-R	GGTGGTGGTGGTGCTCGAGGTCTTCAACGTGGTACTTTCCTTC
271570-F	CAAATGGGTCGCGGATCCAGACCCCAGAAGAAAGGAGGATAC
271570-R	GGTGGTGGTGGTGCTCGAGAGAAGGCCTCGACTGGATTTCAC
267410-F	CAAATGGGTCGCGGATCCGTTAACCTTCGAAGTGATGTCAGCC
267410-R	GGTGGTGGTGGTGCTCGAGTGCGTCGATGGAATCACTTTG
271580-F	CAAATGGGTCGCGGATCCACAGCCCTGGCTGACGCTGTTAC
271580-R	GGTGGTGGTGCTCGAGTGCTCGCAGTGATTCGATAGCATAAGACTC

### 2.2 Sources of sheep serum samples

The 1,350 sheep serum samples used in this epidemiological investigation were sourced from the Hubei Provincial Animal Disease Prevention and Control Center. The negative control serum for *Toxoplasma gondii*, as well as the positive sera for cyst infection, oocyst infection, and *Haemonchus contortus* infection in goats, were provided by the Parasitology Laboratory of Huazhong Agricultural University.

### 2.3 Recombinant protein expression and purification

The recombinant plasmid carrying the target fragment was transformed into *E. coli* BL21 (DE3) competent cells. A single positive colony was selected and grown overnight in LB medium with antibiotics. The culture was then diluted in fresh medium and allowed to grow to the logarithmic phase. Protein expression was induced with 1 mmol/L IPTG, while a non-induced culture was maintained as a control. After induction, cells were harvested and lysed. Protein expression was verified by SDS-PAGE (23). Following condition optimization, the target protein was purified using affinity chromatography.

### 2.4 Validation of ideal antigen

The purified proteins were used as antigens and incubated with standard positive sera from sheep infected with *Toxoplasma gondii* oocysts and positive sera from sheep infected with tissue cysts. The standard positive serum from sheep infected with *Toxoplasma gondii* oocysts was prepared in our laboratory. Briefly, sheep were experimentally infected with *Toxoplasma gondii* oocysts, and the serum was collected and stored for subsequent use. Similarly, the standard positive serum for tissue cysts was obtained using the same experimental infection protocol. Western blot analysis was employed to verify the oocyst-specificity of the proteins. The ideal antigen was selected based on its ability to react specifically with sera from oocyst-infected sheep, while showing no reactivity with sera from cyst-infected sheep or negative control sera.

### 2.5 Construction of the Tg267410-iELISA method

#### 2.5.1 Antigen coating concentration and serum primary antibody titration

The TGME49_267410 antigen was coated onto enzyme-linked immunosorbent assay (ELISA) strips (BIOFIL, Wuhan) at concentrations of 0.25, 0.5, 1, 2, 4, 6, 8, and 16 μg/mL. Positive and negative sera were diluted at ratios of 1:25, 1:50, 1:100, 1:200, 1:400, and 1:800. The corresponding combinations were set up with replicates for each condition. ELISA was performed, and the optical density (OD) at 630 nm was measured using a microplate reader (Bio-Tech Company, USA). The P/N ratio (the OD value of positive serum divided by that of negative serum) was calculated for each antigen concentration (24). By considering the antigen dilution concentration, serum dilution ratios, and the P/N values, the optimal antigen coating concentration and serum primary antibody concentration were selected.

#### 2.5.2 Optimization of reaction conditions for the Tg267410-iELISA method

To further refine the *Tg*267410-iELISA method, we systematically optimized various reaction conditions based on the preliminary checkerboard titration results. The optimization process included the following parameters: blocking concentration, blocking time, incubation time of the test serum, secondary antibody concentration and incubation time, and substrate reaction time. Specifically, the blocking concentration was evaluated using BSA at 0.1, 0.5, 1.0, and 2.0%, as well as skim milk at 1.0, 2.5, 5.0, and 20.0%. The blocking time was tested at 30, 45, 60, and 75 min. The incubation time of the test serum was assessed at 15, 30, 45, 60, and 75 min. The secondary antibody concentration was optimized at 1:2000, 1:3000, 1:4000, 1:5000, and 1:6000, with corresponding incubation times of 15, 30, 45, 60, and 75 min. Finally, the substrate reaction time was evaluated at 5, 10, 15, 20, and 25 min. Each condition was tested in triplicate to ensure reproducibility and to determine the optimal reaction conditions for the assay.

#### 2.5.3 Sensitivity and specificity evaluation of the Tg267410-iELISA method

To determine the sensitivity of the *Tg*267410-iELISA method, two positive serum samples were subjected to serial dilutions ranging from 1:25 to 1:3200. The optimized assay conditions were employed to conduct the ELISA, with positive, negative, and blank controls included in each experiment. The highest dilution at which the assay still detected a positive signal was identified as the sensitivity endpoint. To assess the specificity of the *Tg*267410-iELISA method, the assay was performed on positive serum samples from goats infected with *Haemonchus contortus*. The presence of cross-reactivity was evaluated by determining whether false-positive results were obtained.

#### 2.5.4 Clinical sample detection

The optimized *Tg*267410-iELISA method was employed to test 1,350 serum samples collected from sheep in 69 locations across 17 prefecture-level cities in Hubei Province. The same batch of samples was also tested using the GRA1-iELISA method previously established in our laboratory.

#### 2.5.5 Statistical analysis

The significance of differences among experimental groups was assessed by one-way analysis of variance (one-way ANOVA). This method was appropriate as the study design involved a single independent factor with multiple levels, and the data met the assumptions of normality and homogeneity of variances. Upon obtaining a significant overall *F*-test (*p* < 0.05), Duncan’s new multiple range test was employed for post-hoc pairwise comparisons. Duncan’s test was selected for its balanced capacity to limit Type I errors while retaining high sensitivity to detect true differences, a characteristic that makes it well-suited for exploratory studies like the present one. All statistical analyses were performed using GraphPad Prism 8.0 (GraphPad Software Inc., La Jolla, CA, USA).

## 3 Results

### 3.1 Prokaryotic expression of candidate proteins

**Figure 1:**
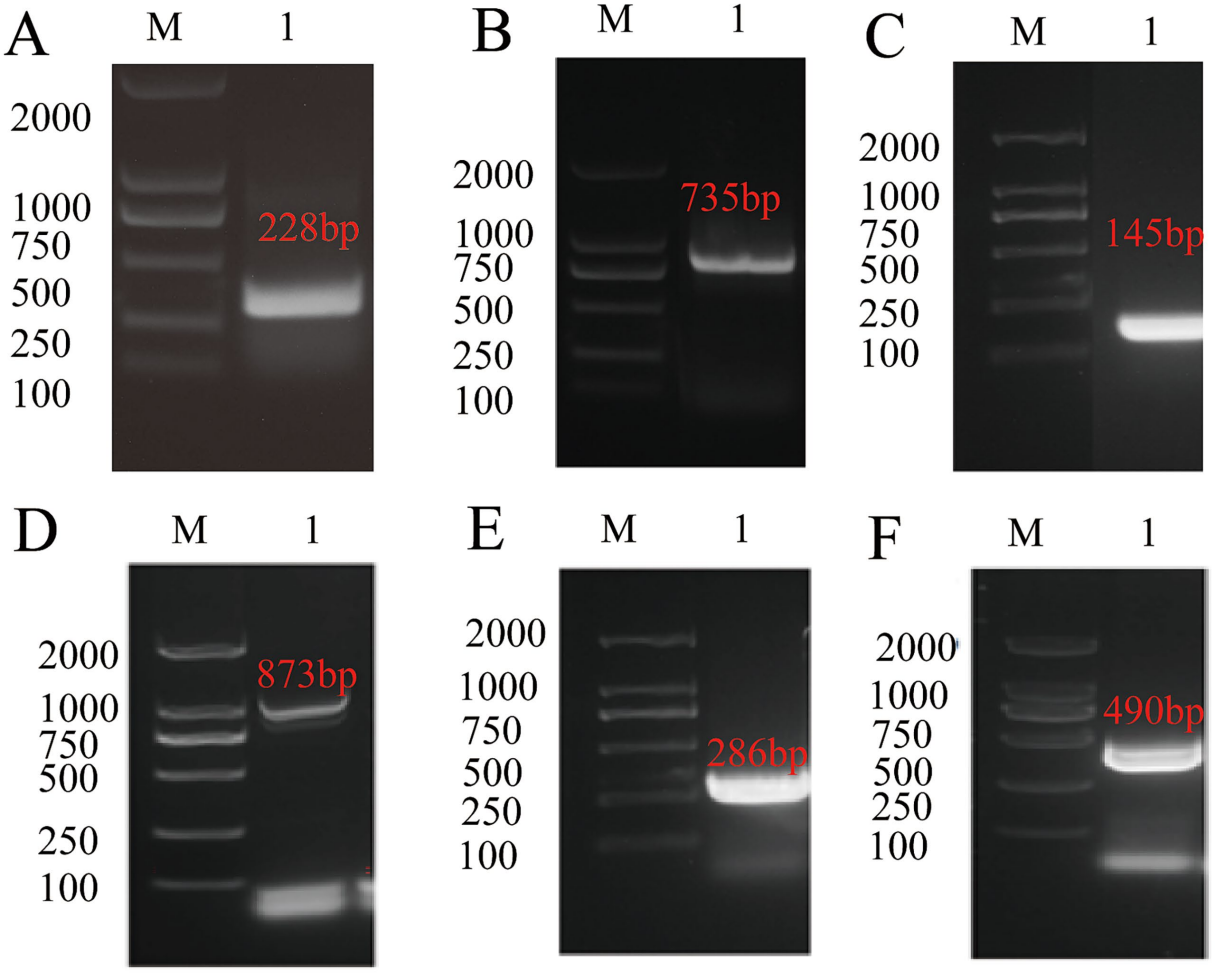
Amplification of target fragments of eight genes including TGME49_267410. M: DNA marker; **(A)** TGME49_313000 fragments; **(B)** TGME49_316550 fragments; **(C)** TGME49_281590 fragments; **(D)** TGME49_292960 fragments; **(E)** TGME49_242600 fragments; **(F)** TGME49_267410 fragments.

Eight genes with higher expression levels during the oocyst stage compared to other stages were identified as potential diagnostic markers for oocysts. Primers were designed to amplify the target fragments of these genes via PCR. The results are shown in Figures 1A–F: the fragments of TGME49_313000 (228 bp), TGME49_316550 (735 bp), TGME49_281590 (145 bp), TGME49_292960 (873 bp), TGME49_242600 (286 bp), TGME49_271570 (384 bp), TGME49_267410 (490 bp), and TGME49_271580 (321 bp) were successfully amplified. The sizes of these fragments were consistent with the predicted values.

### 3.2 Expression of recombinant proteins

**Figure 2:**
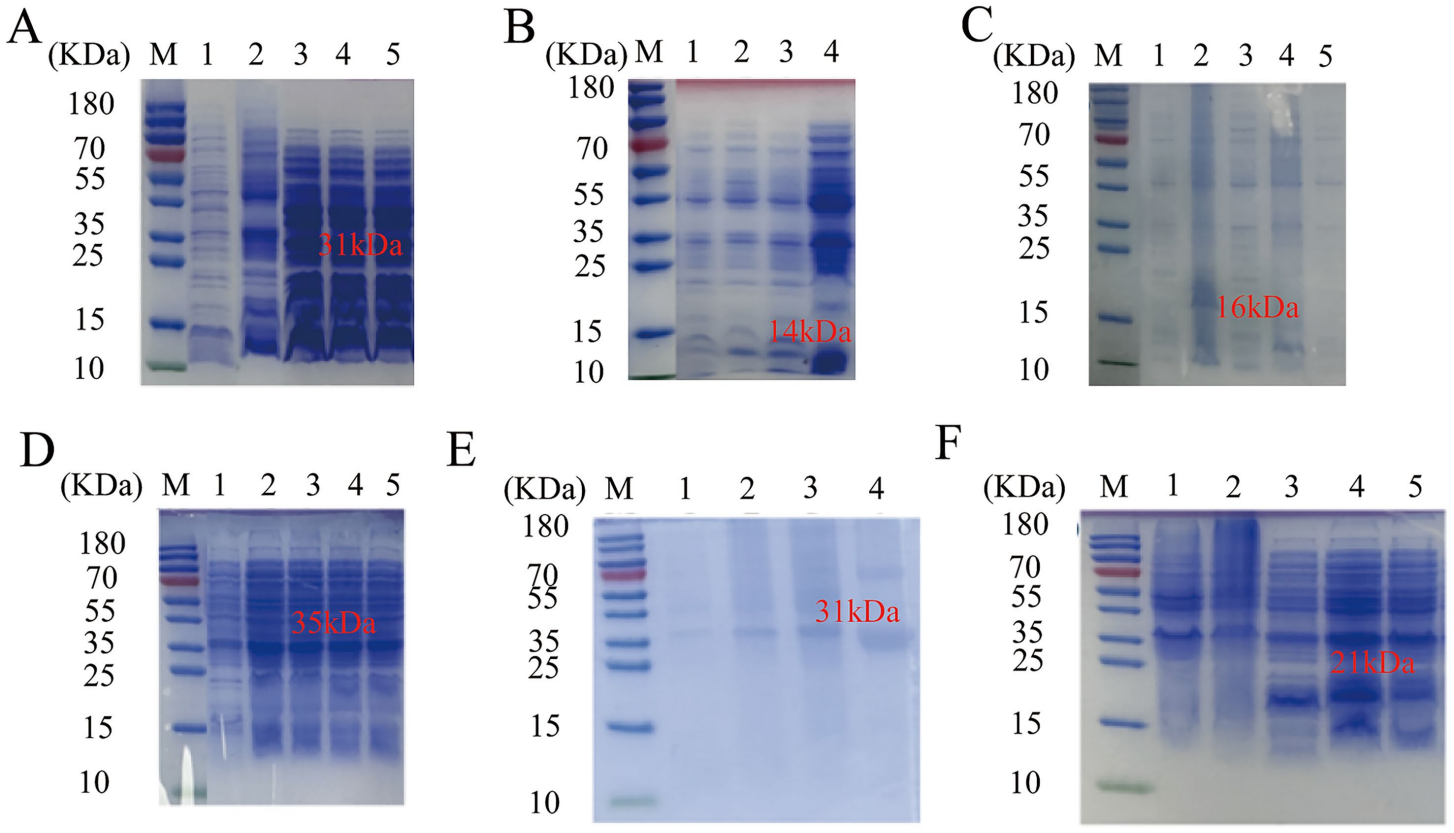
SDS-PAGE analysis of TGME49_267410 and other seven protein expression conditions. M: Protein marker; 1: not induced; 2: 37 °C induced 4–5 h; 3: 16 °C induced 4–5 h; 4: 16 °C induced 16 h; 5: 37 °C induced 16 h; **(A)** TGME49_313000; **(B)** TGME49_316550; **(C)** TGME49_281590; **(D)** TGME49_292960; **(E)** TGME49_242600; **(F)** TGME49_267410.

To facilitate the successful expression of the target proteins, *Escherichia coli* BL21 (DE3) competent cells were employed for prokaryotic expression. Following transformation, bacterial cultures were subjected to shaking incubation and plating. Colonies were then selected and verified to optimize expression conditions. Induction was carried out using IPTG at a concentration of 1:1000 under varying conditions, including different temperatures (37 °C and 16 °C) and induction durations (4–5 h and 16 h). The results are illustrated in Figure 2: TGME49_313000 was successfully expressed at 16 °C with a 4-5-h induction, resulting in a band of approximately 31 kDa (Figure 2A). TGME49_316550 was expressed at 37 °C with a 4–5-h induction, yielding a band of approximately 14 kDa (Figure 2B). TGME49_281590 was expressed at 37 °C with a 4–5-h induction, producing a band of approximately 16 kDa (Figure 2C). TGME49_292960 was expressed at 37 °C with a 4–5-h induction, resulting in a band of approximately 35 kDa (Figure 2D). TGME49_242600 was expressed at 16 °C with a 16-h induction, yielding a band of approximately 31 kDa (Figure 2E). TGME49_267410 was expressed at 16 °C with a 16-h induction, resulting in a band of approximately 21 kDa (Figure 2F). These findings confirmed the successful expression of seven proteins, including TGME49_267410. However, TGME49_271580 failed to be expressed under any of the tested temperature and induction time conditions.

### 3.3 Recombinant protein purification

**Figure 3:**
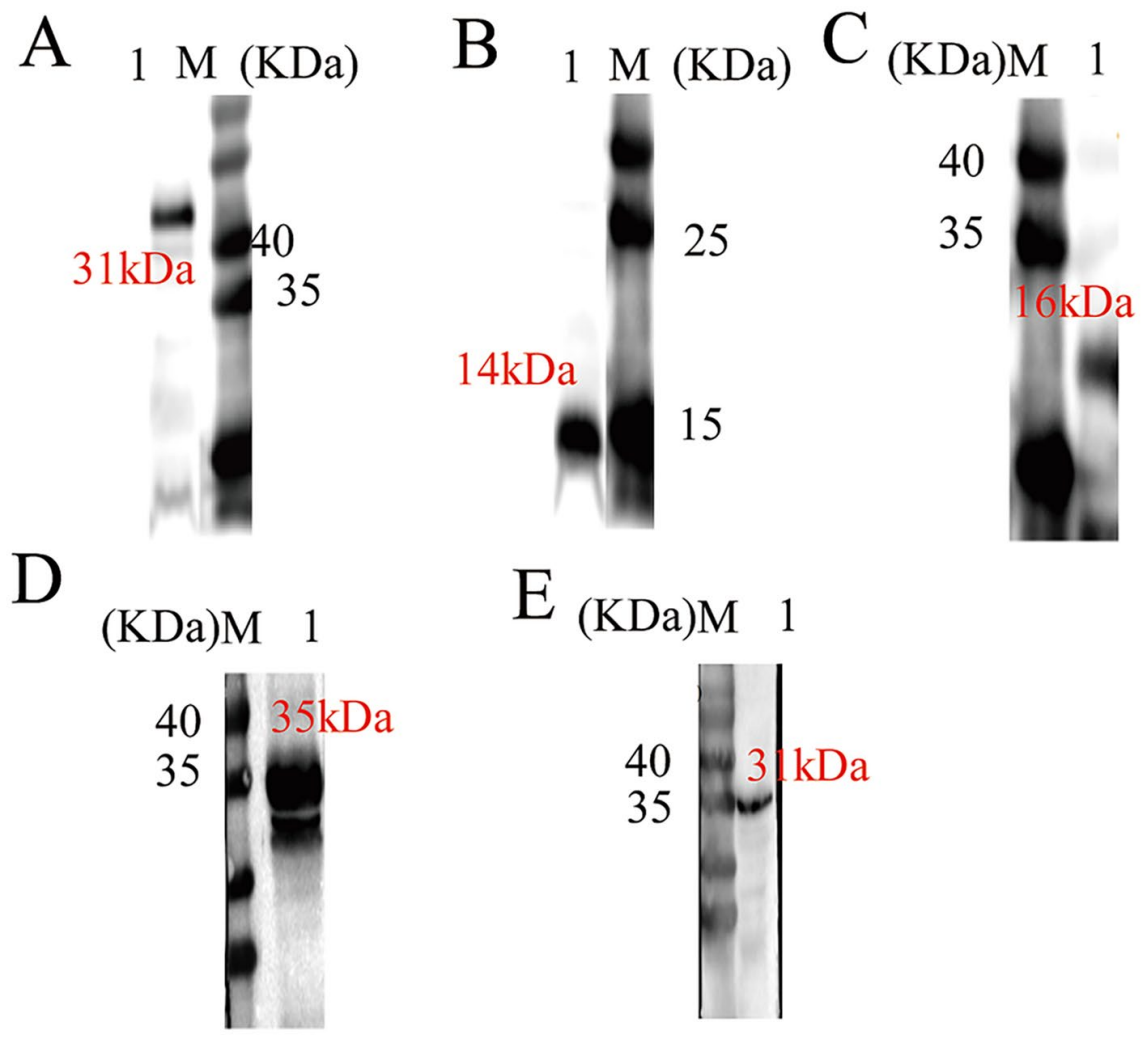
SDS-PAGE analysis and western-blot analysis of seven protein purifications including TGME49_267,410. M: Protein marker; **(A)** TGME49_313000 (31 kDa); **(B)** TGME49_316550 (14 kDa); **(C)** TGME49_281590 (16 kDa); **(D)** TGME49_292960 (35 kDa); **(E)** TGME49_242600 (31 kDa).

The induced bacterial lysates were analyzed by SDS-PAGE and Western blotting. As shown in Figures 3A–E, the protein TGME49_31300 exhibited a corresponding band at approximately 31 kDa, TGME49_316550 at approximately 14 kDa, TGME49_281590 at approximately 16 kDa, TGME49_292960 at approximately 35 kDa, TGME49_242600 at approximately 31 kDa, TGME49_271570 at approximately 18 kDa, and TGME49_267410 at approximately 21 kDa. These results demonstrate that all seven recombinant proteins were successfully purified under different induction conditions.

### 3.4 Identification of diagnostic antigens

**Figure 4:**
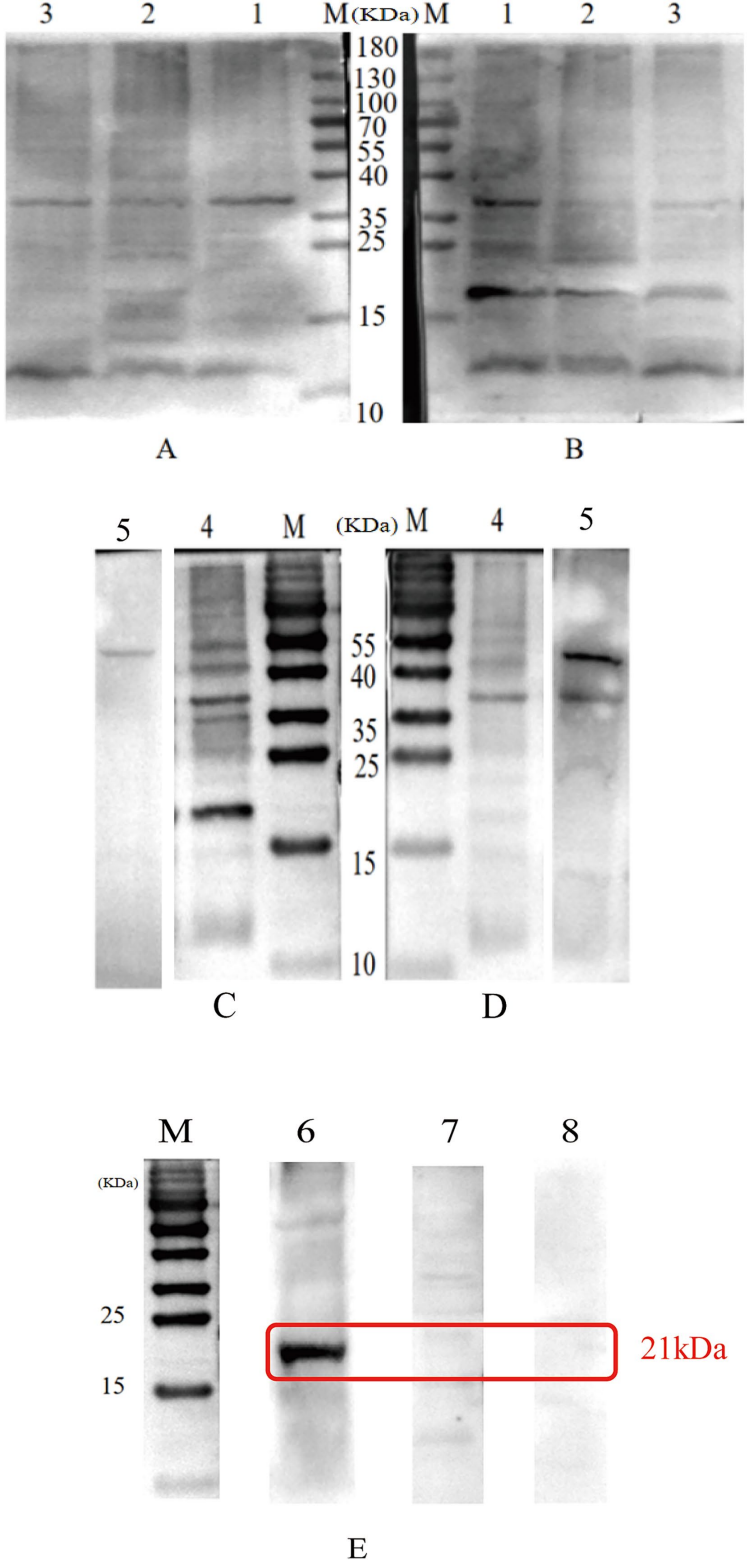
Western-blot analysis of the results of positive serologic identification of oocysts and tissue cyst infection of six proteins including TGME49_313000. M: Protein marker; **(A)** Sheep cystic serum incubation; **(B)** Sheep oocyst serum incubation; **(C)** Sheep oocyst serum incubation; **(D)** Sheep cystic serum incubation; 1: TGME49_313000; 2: TGME49_316550; 3: TGME49_281590; 4: TGME49_292960; 5: TGME49_242600. **(E)** Western-blot analysis of the results of TGME49_267410 protein reacting with positive and negative sera of oocysts and tissue cyst infections. M: Protein marker; 6: Sheep oocyst serum incubation; 7: Sheep cystic serum incubation; 8: Sheep negative serum incubation.

To identify diagnostic antigens, the seven purified recombinant proteins were used as antigens to incubate with sera from sheep with different sources of infection. The specificity of these proteins was then validated using Western blot analysis. The results showed that six proteins, namely TGME49_313000, TGME49_316550, TGME49_281590, TGME49_292960, TGME49_271570, and TGME49_242600, failed to serve as specific diagnostic markers for ELISA-based detection of antibodies against oocyst infection in sheep (Figures 4A–D). In contrast, only one protein, TGME49_267410, exhibited the ability to specifically distinguish between sera from sheep infected with oocysts and those infected with tissue cysts. Notably, this protein did not react with negative sera (Figure 4E). These results indicate that TGME49_267410 was successfully identified as a diagnostic antigen for oocyst infection in sheep.

### 3.5 Optimization of conditions, sensitivity, specificity, and stability experiments

**Figure 5:**
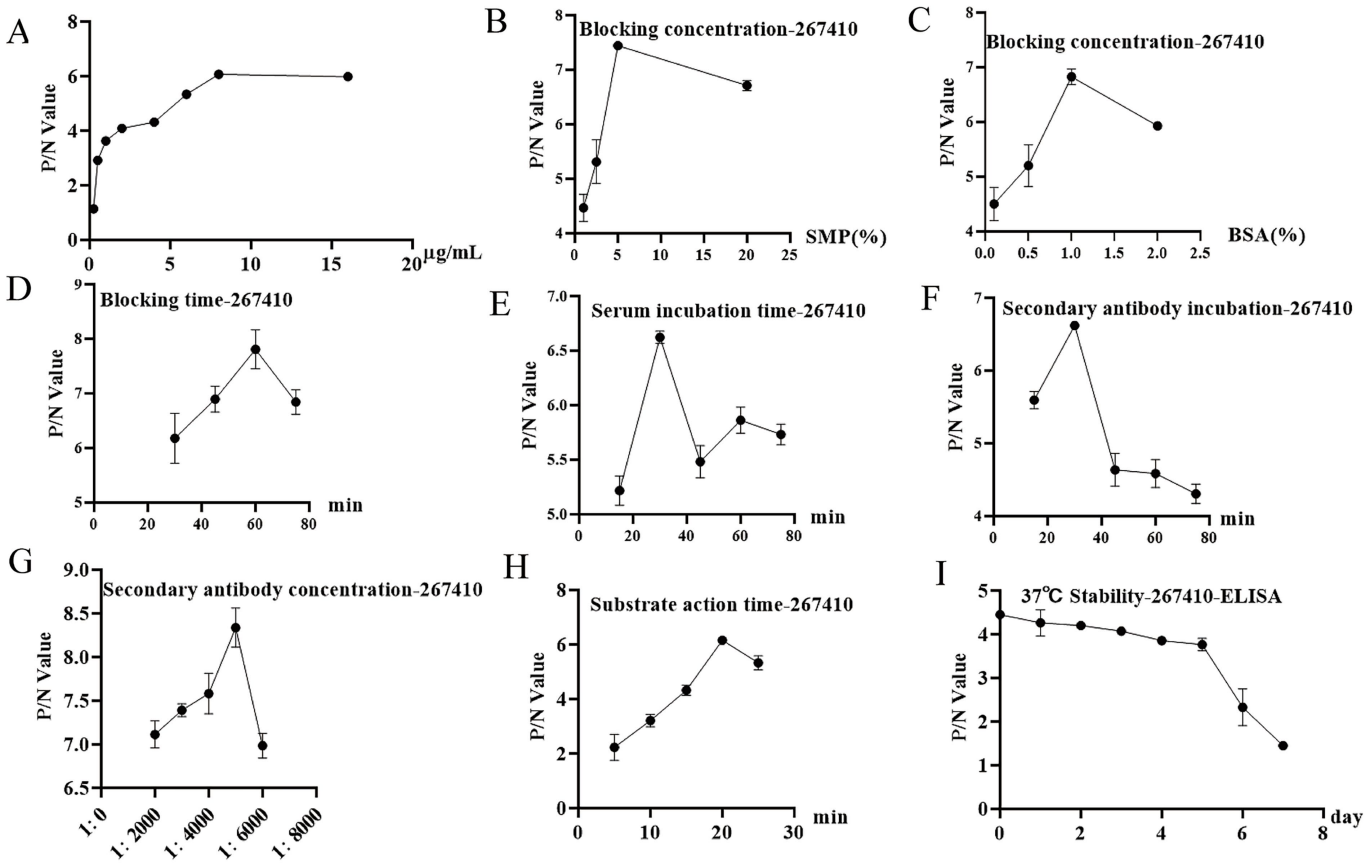
Analysis of TGME49_267410 protein square array titration results. **(A)** The optimal serum dilution of TGME49_267410 protein is 1:100, and this panel **(A)** only shows the P/N values corresponding to the individual tissue cyst infection concentrations at 1:100 dilution. **(B,C)** Determination of the optimal closure solution for TGME49_267410 protein and its concentration. **(D)** Determination of the blocking time of TGME49_267410 protein. **(E,F)** Determination of TGME49_267410 protein serum incubation time and secondary antibody incubation time. **(G,H)** Determination of TGME49_267410 protein secondary antibody incubation concentration and color development time. **(I)** Stability test results of TGME49_267410 protein at 37 °C.

To the results of the checkerboard titration showed that the optimal coating concentration of the protein for the *Tg*267410-iELISA was 8 μg/mL, with a serum dilution of 1:100, corresponding to the maximum OD630 P/N value (Figure 5A). Based on these results, further reaction conditions were optimized using TGME49_267410 as the coating antigen. The optimal blocking concentration was determined to be 5% skimmed milk powder (Figures 5B,C), with an optimal blocking time of 60 min (Figure 5D). The optimal incubation times for serum and secondary antibody were 30 min each (Figures 5E,F), and the optimal dilution ratio for the enzyme-labeled secondary antibody was 1:5000 (Figure 5G). The optimal color development time was 20 min (Figure 5H). Subsequently, the optimized ELISA conditions were used to determine the cut-off value for positive and negative results. Twenty sheep sera, previously confirmed as negative by MAT and GRA1-iELISA, were tested using the *Tg*267410-iELISA, with positive, negative, and blank controls included. The statistical results, as shown in Table 2, revealed that the average S/N value (*x*) of the 20 serum samples was 1.813, with a standard deviation (SD) of 0.459. The cut-off value was calculated as *x* + 3SD = 3.19. This value is based on the statistical principle that *x* + 3SD represents a 99% confidence interval. Therefore, the diagnostic cut-off value for this method was set at 3.19. Samples with an S/N value ≥3.19 were considered positive, while those with an S/N value <3.19 were considered negative.

**Table 2:** Negative serum test results for goats.

20 Goat sera										
OD_630_	0.230	0.162	0.160	0.163	0.153	0.154	0.162	0.154	0.161	0.158
	0.351	0.160	0.166	0.165	0.183	0.172	0.162	0.170	0.171	0.162
S/N	2.375	1.667	1.653	1.68	1.574	1.577	1.67	1.587	1.66	1.63
	3.622	1.65	1.708	1.704	1.883	1.773	1.67	1.749	1.766	1.667
Positive control 0.83 (8.55)				Negative control 0.097 (1)			Blank control 0.065 (0.67)			

To evaluate the sensitivity of the *Tg*267410-iELISA method, two positive sheep sera were serially diluted at eight gradient levels (25-, 50-, 100-, 200-, 400-, 800-, 1600-, and 3,200-fold), and the *Tg*267410-iELISA was repeated under consistent conditions. As shown in Table 3, the P/N value remained above the cut-off value when the serum dilution reached 1:200, indicating a positive result. This demonstrates that the *Tg*267410-iELISA method has high sensitivity. To assess the specificity of the *Tg*267410-iELISA method, cross-reactivity with *Haemonchus contortus* infection was evaluated using positive goat sera from our laboratory. The results, as shown in Table 4, indicated no reactivity with *Haemonchus contortus* positive sera, confirming the excellent specificity of the *Tg*267410-iELISA method. To evaluate the stability of the *Tg*267410-iELISA method at 37 °C, seven coated microplate strips with a protein concentration of 8 μg/mL were blocked and stored in a 37 °C incubator, with daily sampling at the same time. The results showed that, in the absence of additional stabilizers, the strips remained functional and could detect positive signals up to the fifth day (Figure 5I). This indicates that the *Tg*267410-iELISA method exhibits good stability at 37 °C.

**Table 3:** Sensitivity test results.

Sample name	Dilution ratio							
Not Applicable	1:25	1:50	0.111111111	0.180555556	0.319444444	0.597222222	1.152777778	2.263888889
Serum sample 1	0.641	0.609	0.565	0.472	0.348	0.27	0.168	0.121
	-5.859	-5.567	-5.168	-4.314	-3.18	-2.47	-1.534	-1.11
Serum sample 2	0.62	0.548	0.439	0.355	0.228	0.144	0.142	0.122
	-5.674	-5.015	-4.018	-3.244	-2.085	-1.314	-1.296	-1.116
Positive control 0.739 (6.76)			Negative control 0.109 (1)			Blank control 0.062 (0.57)		

**Table 4:** Specificity test results.

Serum type	*Haemonchus contortus*	
OD_630_ ± SD	0.365 ± 0.096	
S/N	2.251	
Cross-reactivity	No	
Positive control 0.929 (5.725)	Negative control 0.16 (1)	Blank control 0.0897 (0.552)

### 3.6 Clinical sample detection results

**Figure 6:**
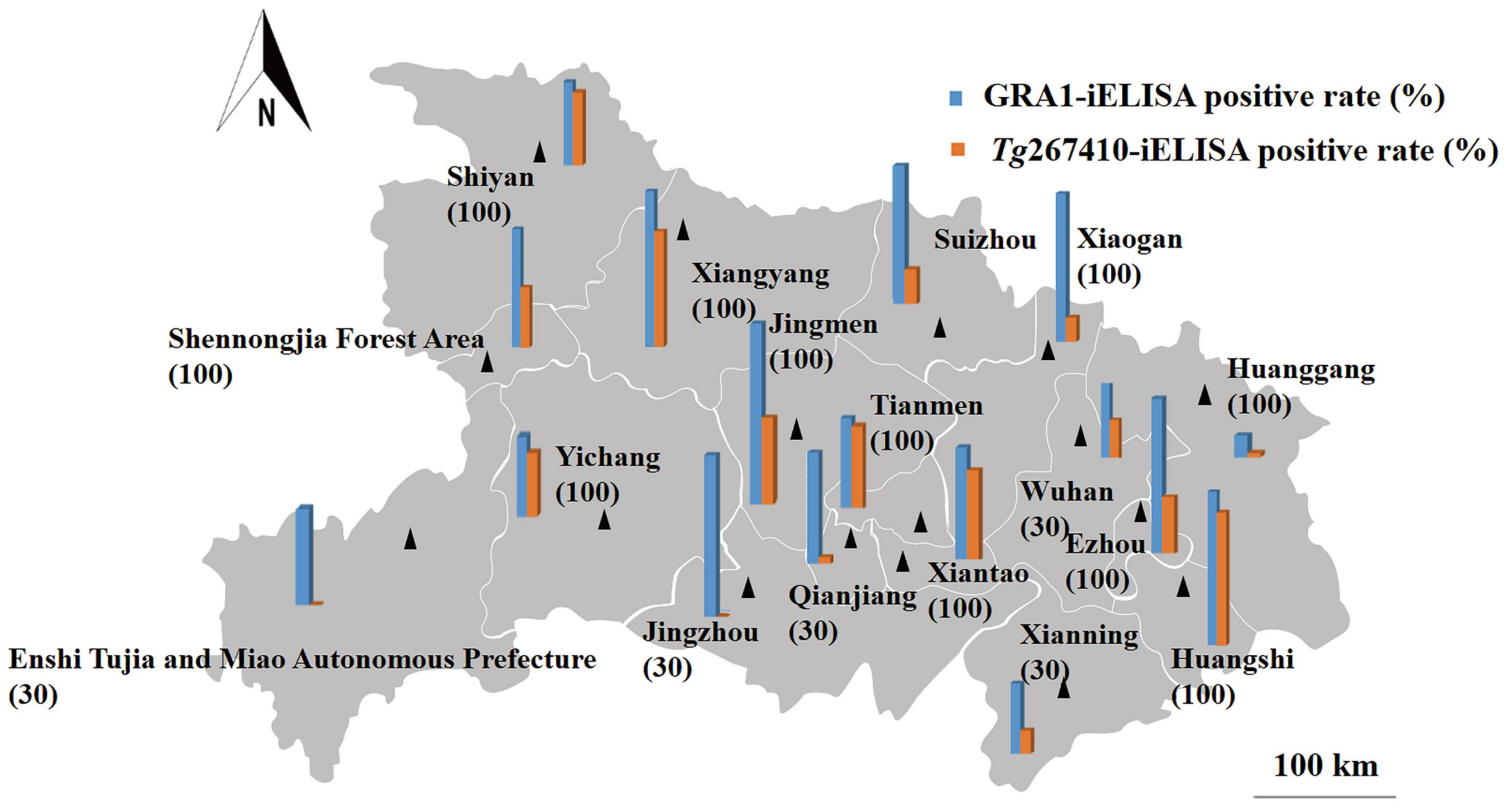
Geographic distribution of positive serum samples in various cities of Hubei province. Note: The colors of the map from dark to light indicate the different positivity rates of sheep infected with *Toxoplasma* oocysts.

**Figure 7:**
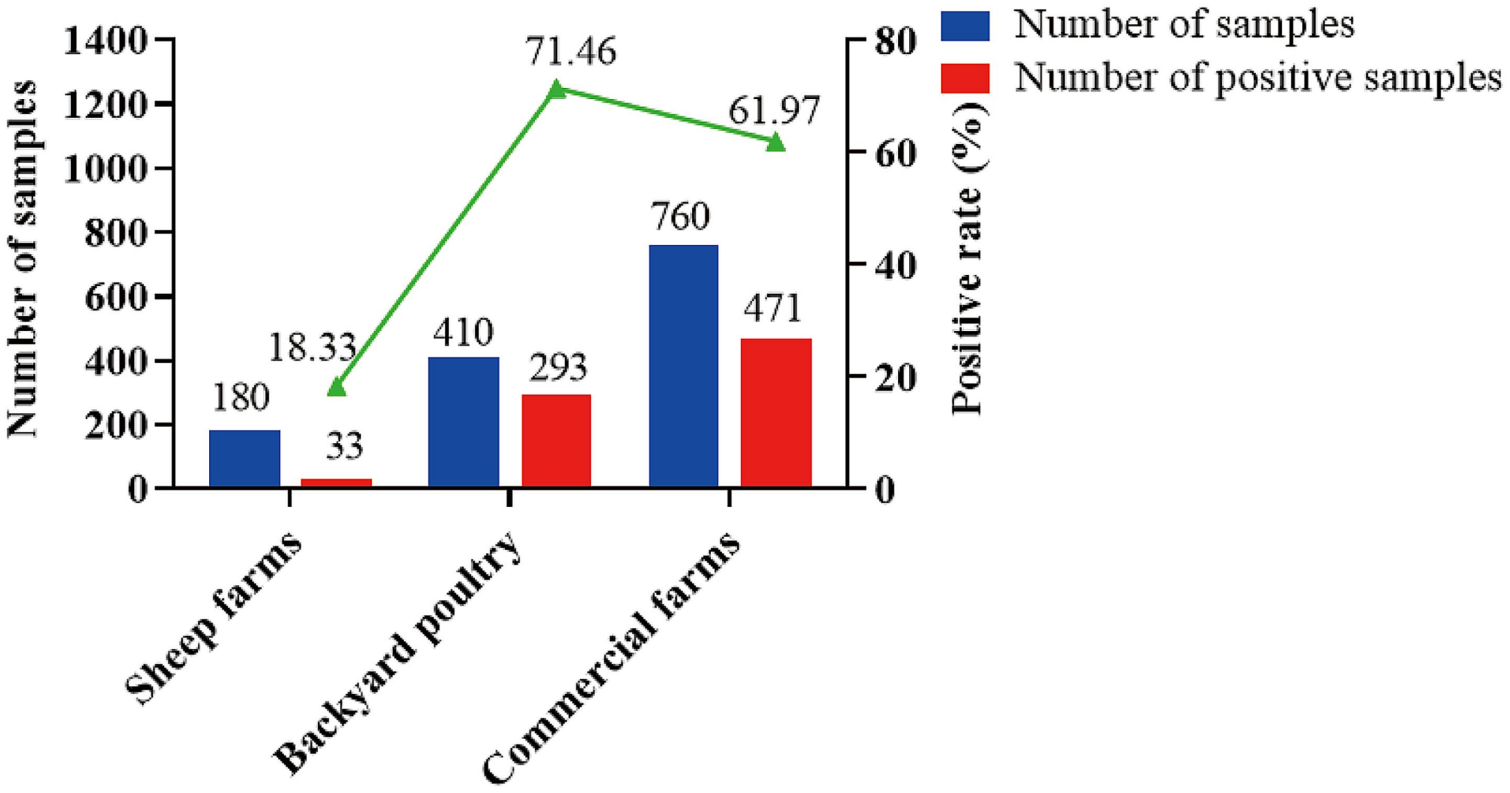
Detection of positive *Toxoplasma gondii* oocysts in sheep infected with *Toxoplasma gondii* in different field types.

A total of 1,350 sheep serum samples from 17 prefecture-level cities in Hubei Province were tested using the optimized *Tg*267410-iELISA method. Concurrently, these samples were also tested using the previously established GRA1-iELISA method in our laboratory. As shown in Table 5, Figure 6, the average positive rate of *Toxoplasma gondii* oocyst infection detected by the *Tg*267410-iELISA method was approximately 28.0% (378/1,350), while the average positive rate of overall *T. gondii* infection detected by the GRA1-iELISA method was approximately 59.0% (797/1,350). In Hubei Province, the overall oocyst infection rate was 47.4%, and the cyst infection rate was 52.6%. Among different types of sheep farms, the lowest positive rate of oocyst infection (12.2%) was observed in breeding farms, while the highest positive rate (39.9%) was found in commercial fattening farms (Figure 7).

**Table 5:** Testing of clinical samples in Hubei province.

Method	GRA1-iELISA			Total
*Tg*267410-iELISA		Positive	Negative	
	Positive	378	0	378
	Negative	419	553	972
Total		797	553	1,350

## 4 Discussion

Toxoplasmosis control is as challenging as the control of diseases caused by other parasites. Notably, as the number of sheep raised in China continues to grow annually, the prevalence of *Toxoplasma gondii* infection in this livestock population remains persistently high—further exacerbating the aforementioned control challenges in the Chinese sheep industry (25–33). Therefore, establishing a specific detection method to distinguish *T. gondii* oocyst infection in sheep is of great importance (19). The diagnosis of toxoplasmosis is challenging due to the complex life cycle of the parasite and the unknown sources of infection (34–41). Given the simplicity and scalability of ELISA methods, many researchers have developed various antigen-based ELISA assays to detect *T. gondii* antibodies in different livestock species. For example, the sporozoite-specific embryogenesis-related protein (ERP) has been used to differentiate oocyst infections from cyst infections (21). However, existing kits for distinguishing *T. gondii* cyst and oocyst infections still have limitations in terms of specificity and sensitivity (42). Therefore, selecting an ideal antigen to develop a specific ELISA method for detecting *T. gondii* oocyst infection in sheep is crucial.

In this study, eight genes were selected for investigation primarily because they are highly expressed during the oocyst stage but are either lowly expressed or not expressed during the cyst stage, indicating their potential as diagnostic markers for oocyst infection. The results showed that only the protein TGME49_267410 reacted with sera from sheep infected with oocysts but did not react with sera from sheep infected with cysts or with negative sera. This indicates that the TGME49_267410 protein can distinguish the route of *T. gondii* oocyst infection in sheep, allowing for the establishment of the *Tg*267410-iELISA method. Using the optimized Tg267410-iELISA method, 1,350 sheep serum samples from 17 prefecture-level cities in Hubei Province were tested. Additionally, the same batch of samples was tested using the GRA1-iELISA method previously established in our laboratory. The results showed that the overall positive rate of *T. gondii* infection detected by the GRA1-iELISA method in Hubei Province was 59.0%, while the overall positive rate of *T. gondii* oocyst infection detected by the Tg267410-iELISA method was approximately 28.0%. The positive rates of oocyst infection in sheep varied significantly among different cities in Hubei Province, likely due to differences in climate and control measures. Among the 1,350 sheep serum samples, 797 were positive for *T. gondii* infection, with an oocyst infection rate of approximately 47.4% (378/797) and a cyst infection rate of approximately 52.6% (419/797). This suggests that both oocyst and cyst infections are present in sheep in this batch of samples from Hubei Province. The high proportion of cyst infections may be due to sheep ingesting rodent carcasses or contaminated feed during grazing. However, the nearly equal ratio of cyst to oocyst infections (approximately 1:1) in Hubei Province indicates that both types of infections should be equally prioritized in the prevention and control of *T. gondii i*n sheep.

The prevalence of *Toxoplasma gondii* oocyst infection varies significantly among different types of sheep farms. The breeding farms had the lowest positive rate of *T. gondii* oocyst infection at approximately 12.2%, followed by small-scale backyard farms at 12.9%. In contrast, commercial fattening farms exhibited the highest positive rate of oocyst infection at 39.9%. Previous studies have shown that *T. gondii* oocysts have a higher survival rate in warm and humid soil, whereas their survival rate is lower in colder climates (43, 44). Breeding farms, which typically have a large scale of over a thousand animals, often feature stable temperature and humidity conditions, as well as comprehensive automated control systems. Research has indicated that good management practices can significantly reduce the infection rate of *T. gondii* (45–49). Currently, most large-scale farms in Hubei Province have well-implemented management measures, including rodent control, cat prohibition, and maintaining clean and controlled breeding conditions. These measures contribute to the relatively low infection rate of 12.2% in breeding farms, which is significantly lower than that in commercial fattening farms (39.9%). The high positive rate of oocyst infection in commercial fattening farms (39.9%) suggests that the current prevention and control measures for *T. gondii* oocyst infection in these farms may not be sufficient.

This study has several limitations. First, the reliance on a single antigen may constrain the generalizability of our findings. Second, future studies should include sheep serum samples from diverse geographical regions beyond Hubei Province to evaluate potential regional biases. Additionally, extending this research to other ruminant species will be essential to assess the broader applicability and diagnostic performance of the method.

In summary, this study has established a Tg267410-iELISA method that can specifically detect the source of *Toxoplasma gondii* infection in sheep (i.e., via the oocyst route), and it has demonstrated good sensitivity. When used in conjunction with the GRA1-iELISA method previously developed in our laboratory, it can determine the specific mode of *T. gondii* infection in sheep. This combination allows for more accurate detection and targeted prevention of ovine toxoplasmosis.

## Data Availability

The datasets presented in this study can be found in online repositories. The names of the repository/repositories and accession number(s) can be found in the article/Supplementary material.
