# Supplementary material for: Establishment of an ELISA for detecting oocyst-derived Toxoplasma gondii infections in sheep
Source: Front Vet Sci. 2026 Jan 14;12:1674011. doi: 10.3389/fvets.2025.1674011 (PMC12853370; doi:10.3389/fvets.2025.1674011)
Supplement: Supplementary file 1 [file Table_1.DOCX]

Table S1 The P/N values corresponding to each coating concentration of TGME49_267410 protein at a dilution of 1:100

| Protein coating concentration | P/N index |
| --- | --- |
| 0.25 | 1.13 |
| 0.50 | 2.92 |
| 1.00 | 3.63 |
| 2.00 | 4.09 |
| 4.00 | 4.32 |
| 6.00 | 5.34 |
| 8.00 | 6.07 |
| 16.00 | 5.98 |

Table S2 The optimal concentration of Skimmed Milk Powder (SMP) for the protein TGME49_267410

| SMP(%) | P/N index | | |
| --- | --- | --- | --- |
| 1.00 | 4.75 | 4.34 | 4.31 |
| 2.50 | 5.75 | 4.96 | 5.23 |
| 5.00 | 7.39 | 7.44 | 7.49 |
| 20.00 | 6.74 | 6.61 | 6.79 |

Table S3 The optimal concentration of Bovine Serum Albumin (BSA) for the protein TGME49_267410

| BSA（%） | P/N index | | |
| --- | --- | --- | --- |
| 0.10 | 4.85 | 4.39 | 4.27 |
| 0.50 | 5.64 | 5.00 | 4.96 |
| 1.00 | 6.83 | 6.67 | 6.97 |
| 2.00 | 5.86 | 5.99 | 5.94 |

Table S4 The optimal blocking time for the blocking agent required by the protein TGME49_267410

| Time(min) | P/N index | | |
| --- | --- | --- | --- |
| 30.00 | 6.58 | 6.28 | 5.68 |
| 45.00 | 7.11 | 6.93 | 6.64 |
| 60.00 | 7.84 | 7.44 | 8.15 |
| 75.00 | 6.78 | 6.65 | 7.09 |

Table S5 The optimal incubation time for the primary antibody required by the protein TGME49_267410

| Time(min) | P/N index | | |
| --- | --- | --- | --- |
| 15.00 | 5.21 | 5.36 | 5.09 |
| 30.00 | 6.56 | 6.65 | 6.65 |
| 45.00 | 5.58 | 5.56 | 5.31 |
| 60.00 | 5.79 | 5.79 | 6.00 |
| 75.00 | 5.76 | 5.81 | 5.63 |

Table S6 The optimal incubation time for the secondary antibody required by the protein TGME49_267410

| Time(min) | P/N index | | |
| --- | --- | --- | --- |
| 15.00 | 5.73 | 5.49 | 5.57 |
| 30.00 | 6.56 | 6.65 | 6.65 |
| 45.00 | 4.87 | 4.42 | 4.62 |
| 60.00 | 4.76 | 4.38 | 4.62 |
| 75.00 | 4.15 | 4.38 | 4.39 |

Table S7 The optimal dilution ratio for the secondary antibody required by the protein TGME49_267410

| Ratio | P/N index | | |
| --- | --- | --- | --- |
| 1：2000 | 7.10 | 7.27 | 6.96 |
| 1：3000 | 7.39 | 7.46 | 7.32 |
| 1：4000 | 7.84 | 7.39 | 7.51 |
| 1：5000 | 8.28 | 8.59 | 8.15 |
| 1：6000 | 7.13 | 6.97 | 6.85 |

Table S8 The optimal color development time for the secondary antibody required by the protein TGME49_267410

| Time(min) | P/N index | | |
| --- | --- | --- | --- |
| 10.00 | 1.95 | 1.96 | 1.79 |
| 15.00 | 3.13 | 3.05 | 3.03 |
| 20.00 | 4.30 | 4.24 | 4.12 |
| 25.00 | 6.05 | 6.20 | 6.14 |
| 30.00 | 5.12 | 5.26 | 5.26 |

Table S9 The optimal usage time of TGME49_267410 protein under 37℃ conditions

| Days | P/N index | | |
| --- | --- | --- | --- |
| 0 | 4.50 | 4.39 | 4.46 |
| 1 | 4.51 | 3.93 | 4.35 |
| 2 | 4.21 | 4.25 | 4.15 |
| 3 | 4.07 | 4.09 | 4.06 |
| 4 | 3.80 | 3.89 | 3.87 |
| 5 | 3.82 | 3.88 | 3.60 |
| 6 | 2.04 | 2.12 | 2.81 |
| 7 | 1.40 | 1.43 | 1.51 |

Table S10 *Toxoplasma gondii* infection in sheep by city in Hubei province

| Sample source | The number of sheep farms | The number of test samples | The positive rate of GRA1-iELISA (%) | The positive rate of *Tg*267410-iELISA  (%) |
| --- | --- | --- | --- | --- |
| Wuhan | 3 | 30 | 46.67 | 23.33 |
| Huangshi | 5 | 100 | 96.00 | 83.00 |
| Shiyan | 5 | 100 | 40.00 | 35.00 |
| Jingzhou | 3 | 30 | 73.33 | 0.00 |
| Yichang | 1 | 100 | 25.00 | 20.00 |
| Xiangyang | 5 | 100 | 97.00 | 72.00 |
| Ezhou | 5 | 100 | 78.00 | 29.00 |
| Jingmen | 5 | 100 | 96.00 | 46.00 |
| Huanggang | 5 | 100 | 10.00 | 2.00 |
| Xiaogan | 5 | 100 | 86.00 | 14.00 |
| Xianning | 3 | 30 | 40.00 | 13.33 |
| Xiantao | 5 | 100 | 60.00 | 48.00 |
| Qianjiang | 3 | 30 | 56.67 | 3.33 |
| Shennongjia Forestry District | 5 | 100 | 77.00 | 39.00 |
| Enshi Tujia and Miao Autonomous Prefecture | 3 | 30 | 33.33 | 0.00 |
| Tianmen | 3 | 100 | 33.00 | 30.00 |
| Suizhou | 5 | 100 | 63.00 | 16.00 |
| Total | 69 | 1350 | 59.04 | 28.00 |

Table S11 Positive rate of *Toxoplasma gondii* infection via oocysts in different types of sheep

| Type | Number of samples | Number of positive samples | Positive rate of oocyst infection |
| --- | --- | --- | --- |
| Sheep farms | 180 | 33 | 18.33 |
| Backyard poultry | 410 | 293 | 71.46 |
| Commercial farms | 760 | 471 | 61.97 |
